# Endovascular management of intermediate-risk pulmonary embolism: evidence, outstanding questions, drivers of utilization, and the horizon

**DOI:** 10.1093/ehjopen/oeaf071

**Published:** 2025-06-04

**Authors:** Arman A Shahriar, Jonathan Paul, Adam Cifu

**Affiliations:** Department of Medicine, The University of Chicago, 5841 S. Maryland Ave, Chicago, IL 60637, USA; Department of Medicine, The University of Chicago, 5841 S. Maryland Ave, Chicago, IL 60637, USA; Section of Cardiology, The University of Chicago, 5841 S. Maryland Ave, Chicago, IL 60637, USA; Department of Medicine, The University of Chicago, 5841 S. Maryland Ave, Chicago, IL 60637, USA; Section of General Internal Medicine, The University of Chicago, 5841 S. Maryland Ave, Chicago, IL 60637, USA

**Keywords:** Pulmonary embolism, venous thromboembolism, endovascular, utilization, evidence-based medicine

## Abstract

In patients with acute intermediate-risk pulmonary embolism (PE) guidelines recommend systemic anticoagulation (Class I; Level A), but intermediate-risk patients are increasingly being treated with adjunctive endovascular (catheter-based) therapies. This review defines outstanding clinical questions, contextualizes completed and ongoing clinical studies, examines plausible drivers of utilization, and anticipates scenarios on the horizon for endovascular therapy in this large and heterogenous subgroup of patients. In intermediate-risk PE, up-front adjunctive systemic thrombolysis reduces haemodynamic deterioration or death, but the small benefit is outweighed by the risk of major bleeding. Endovascular modalities (e.g. ultrasound-assisted catheter directed thrombolysis, mechanical thrombectomy) aim to uphold these benefits while improving upon safety. Since 2014, five devices have entered the market based primarily on single-arm studies demonstrating short-term improvements in surrogate markers of effectiveness (e.g. 48 h reduction in RV/LV ratio). While thousands of patients with intermediate-risk PE (primarily intermediate-*high* risk PE) have been enrolled in prospective studies using these devices, only three small Randomized controlled trials (RCTs) have compared adjunctive endovascular therapy with anticoagulation alone, and none have included patient-centred efficacy endpoints (i.e. mortality or morbidity). In the absence of high-quality evidence or guideline recommendations, rising utilization in intermediate-risk patients may be driven by clinical uncertainty, PE response teams, favourable regulation and reimbursement, industry marketing, and financial incentives for various stakeholders. Three large RCTs are currently enrolling patients to evaluate both short- and long-term patient-centred measures of efficacy as well as safety of adjunct endovascular therapy relative to anticoagulation alone. The results of these trials will provide critical insights by the decade’s end.

## Graphical Abstract

**Graphical Abstract:**
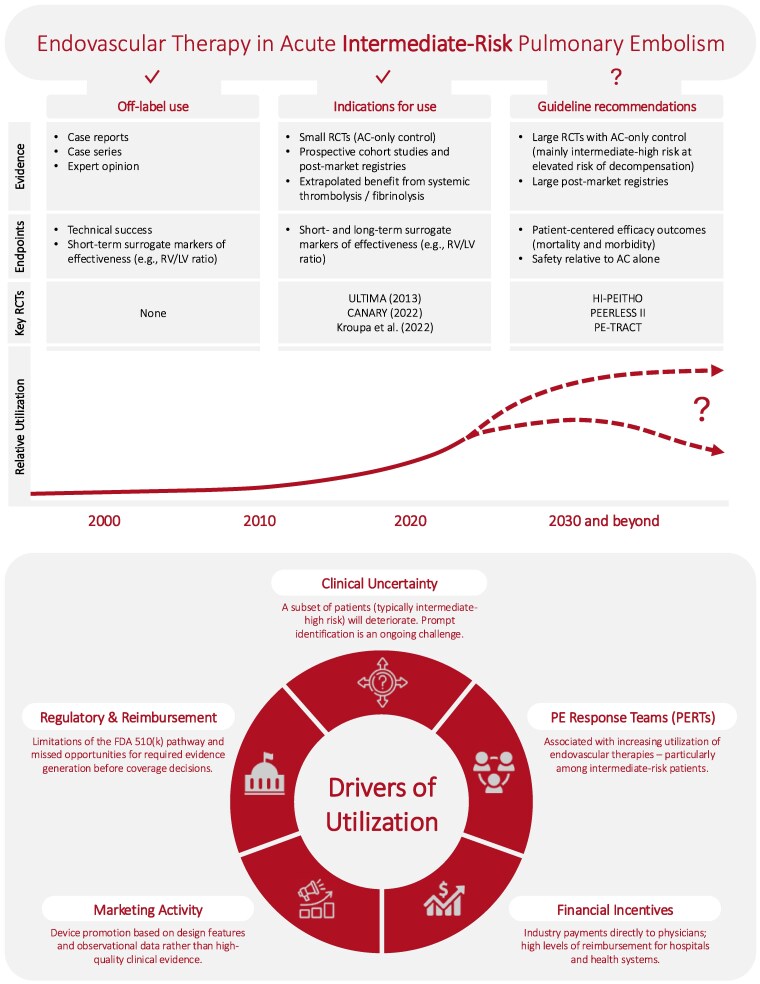

## Introduction

Acute pulmonary embolism (PE) is a leading cause of cardiovascular morbidity and mortality worldwide.^1,2^ The disease presents along a haemodynamic continuum, from small incidentally discovered PE to obstructive shock and circulatory collapse, with most patients presenting somewhere between these two extremes. Anticoagulation remains the cornerstone of all PE management, but adjunctive therapies (e.g. medical, endovascular, and surgical) directed at rapid reperfusion are sometimes warranted.^3,4^

In the past decade, there has been a significant increase in utilization of endovascular (catheter-based) therapies in acute PE^5–9^ following the regulatory authorizations of multiple devices based primarily on small, uncontrolled studies with short-term surrogate endpoints.^10–15^ Endovascular therapies are increasingly being used for patients with haemodynamic instability (i.e. high-risk PE) where immediate reperfusion therapy is indicated,^3^ but also for the much larger and heterogeneous group of haemodynamically stable patients who demonstrate evidence of cardiac dysfunction. Indeed, these intermediate-risk patients (mainly intermediate-high risk) comprise the majority of cases currently being managed with endovascular therapies^5,8^ despite there being no Class I recommendations for their up-front use in this subgroup.^3,4^

Against the backdrop of utilization that has outpaced evidence, this review critically examines the past, present, and future of endovascular therapy in intermediate-risk PE, which is important given the size of the affected population, uncertainty of benefits, and risks introduced by adjunctive procedural management when the standard of care remains medical therapy with anticoagulation alone.

## History and importance

Medical device innovation is vital for advancing cardiovascular medicine, and minimally invasive endovascular therapies offer attractive options for patients. Some therapies, such as transcatheter aortic valve replacement in severe aortic stenosis, provide alternatives to more invasive, surgical procedures. Others, such as percutaneous coronary intervention in stable coronary artery disease, complement medical therapy alone. In acute intermediate-risk PE, endovascular therapy complements medical therapy with systemic anticoagulation.

Randomized controlled trials (RCTs) designed to address meaningful clinical questions with proper controls and patient-centred endpoints (morbidity and mortality) advance medical practice, irrespective of their results. Positive results can shift the standard of care, and negative results can reduce exposure to ineffective or harmful therapy. While cardiology remains a leader in evidence-based medicine, several practices for common cardiovascular conditions have been reversed after landmark RCTs demonstrated no benefit, and in some cases, harm.^16,17^ Still, many cardiology guideline recommendations, including those referencing endovascular therapy for intermediate-risk PE,^3,18^ rely on lower levels of evidence.^19^

## Risk stratification and overview of management

Both American Heart Association (AHA) and European Society of Cardiology (ESC) recommend risk-stratifying patients with PE based on the likelihood of early (30 day) mortality, using a combination of clinical, laboratory, and imaging parameters.^3,4^ The principles of management at either extreme are generally not debated. The ESC defines high-risk PE by haemodynamic instability, which is associated with a 30 day mortality risk exceeding 15%.^20–22^ These patients require immediate adjunctive intervention directed at reperfusion (medical, endovascular, or surgical) to prevent death. By contrast, low-risk PE—characterized by normotension, low clinical severity, and no evidence of cardiac dysfunction—carries a low early mortality risk of <1%^20,23^ and can generally be safely managed with anticoagulation alone in the outpatient setting.

Intermediate-risk patients are haemodynamically stable but exhibit heightened clinical severity and evidence of cardiac dysfunction.^3,4,24^ These patients account for 25–60% of hospitalized PE cases.^20–22,25^ Short-term mortality rates in intermediate-risk PE range from 1–3% in clinical trials^26,27^ to 3–15% in observational studies.^4,20^ According to the ESC, patients with *both* imaging and laboratory evidence of cardiac dysfunction are deemed intermediate-*high* risk. The optimal management of intermediate-risk PE—particularly intermediate-high risk—remains uncertain, as a subset of these patients are at risk of decompensating and may benefit from up-front adjunctive therapy directed at reperfusion.^28^

## Rationale for endovascular therapy in intermediate-risk pulmonary embolism

The foundation of evidence for up-front aggressive treatment of intermediate-risk PE is the landmark Pulmonary Embolism International Thrombolysis trial (PEITHO; 2014) which randomized 1006 patients to receive either systemic thrombolysis and anticoagulation or anticoagulation alone.^26^ The study found a small benefit in its primary efficacy endpoint (composite of 7 day mortality and haemodynamic decompensation; 2.6% vs. 5.6%, *P* = 0.02) that was outweighed by risks of major extracranial (6.3% vs. 1.2%, *P* ≤ 0.001) and intracranial (2.4% vs. 0.2%, *P* = 0.003) haemorrhage. As a result, systemic thrombolysis is not the standard treatment in intermediate-risk PE and remains reserved for those who deteriorate on anticoagulation alone, or high-risk patients.^3^

Even before the PEITHO trial, some thrombolytic catheters were being used off-label within the pulmonary vasculature to treat PE—primarily in high-risk patients with contraindications to systemic thrombolysis.^6,7^ The findings of PEITHO illuminated a need for therapies that could maintain the efficacy profile of systemic thrombolysis while improving upon safety. Endovascular therapy was positioned to address this need.

## Endovascular device authorizations in the USA

**Figure 1:**
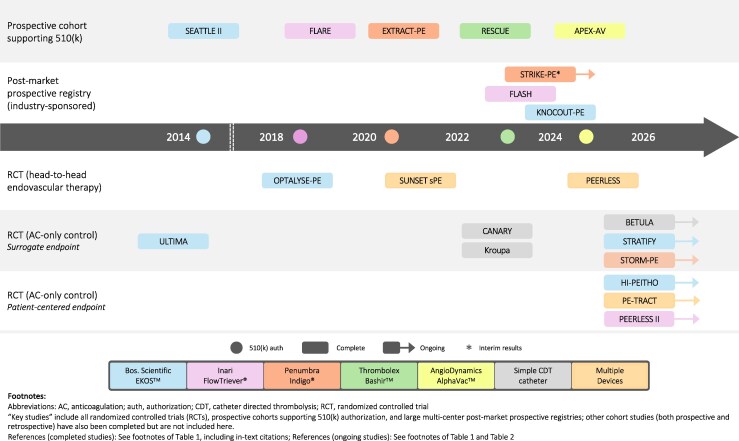
Timeline of key completed *and* ongoing prospective studies of endovascular therapies in intermediate-risk pulmonary embolism, stratified by design and endpoint.

Five endovascular therapies currently hold indications for acute PE in the USA. In 2014, the EKOS^TM^ ultrasound-assisted thrombolysis catheter (Boston Scientific) became the first FDA-authorized endovascular therapy in acute PE. This catheter was initially studied in one RCT of 59 intermediate-risk patients (ULTIMA) and a prospective cohort of 150 intermediate and high-risk patients (SEATTLE II), with both studies demonstrating short-term improvements in the right ventricular/left ventricular (RV/LV) ratio.^14,15^ Thereafter, four analogous prospective cohorts of primarily intermediate-high risk patients examining 48 h improvement in RV/LV ratio—FLARE (2018), EXTRACT-PE (2020), RESCUE (2022), and APEX-AV (2024)—supported FDA authorizations of the FlowTriever® thrombectomy system (Inari Medical, Inc.), Indigo® thrombectomy system (Penumbra, Inc.), Bashir^TM^ pharmaco-mechanical catheter (Thrombolex, Inc.), and the AngioDynamics AlphaVac^TM^ thrombectomy system, respectively (*Figure 1* and *Table 1*).^10–13^ Other endovascular devices are also being used off-label in acute PE.^29^

**Table 1:** Overview of key completed prospective clinical studies of endovascular therapies in intermediate-risk PE, by year of publication

Study^a^	Year^b^	Design	Intervention(s)	N^c^	Patient population (severity)^d^	Primary endpoint	Supported 510(k)
ULTIMA	2013	RCT (AC control)	US-CDT (EKOS^TM^) + AC vs. AC alone	59	Int-high (All RV/LV > 1.0; 80% with trop elevation)	24 h change in RV/LV ratio	Yes
SEATTLE-II	2015	PCS	US-CDT (EKOS^TM^) + AC	150	High (21%); Int-high (79%)	48 h change in RV/LV ratio	Yes
OPTALYSE-PE	2018	RCT (head-to-head)	US-CDT (EKOS^TM^) + AC—4 dosing regimens	101	Int (RV/LV > 1.0)	48 h change in RV/LV ratio; thrombus burden (modified Miller score)	No
FLARE	2018	PCS	MT (FlowTriever®) + AC	106	Majority int-high (All RV/LV > 0.9; 60% trop elevation; 72% proBNP elevation)	48 h change in RV/LV ratio	Yes
EXTRACT-PE	2020	PCS	MT (Indigo®—first gen) + AC	119	Majority int-high (All RV/LV > 0.9; 71% trop elevation; 61% proBNP elevation)	48-h change in RV/LV ratio	Yes
SUNSET sPE	2021	RCT (head-to-head)	US-CDT (EKOS^TM^) + AC vs. Simple CDT + AC	81	Int-high (95%); Int-low (5%)	48 h thrombus burden (modified Miller score)	No
RESCUE	2022	PCS	Pharmaco-mechanical (Bashir^TM^) + AC	109	Majority Int-high (all RV/LV > 0.9; 98% trop or proBNP elevation)	48 h change in RV/LV ratio	Yes
CANARY	2022	RCT (AC control)	CDT + AC vs. AC alone	94	Int-high (100%)	3 month RV/LV ratio > 0.9	No
Kroupa *et al*.	2022	RCT (AC control)	CDT + AC vs. AC alone	23	Int-high (100%)	^e^48 h RV function improvement (2/3 criteria)	No
FLASH	2023	PCS—Registry	MT (FlowTriever®) + AC	800	High (8%); Int-high (83%); Int-low (8%)	48 h change in RV/LV ratio, PA pressures, acute mortality	No
STRIKE-PE (Interim)	2023; ongoing	PCS—Registry	MT (Indigo®—first/second gen) + AC	150	High (5%); Int (95%)	48 h change in RV/LV ratio	No
KNOCOUT-PE	2024	PCS—Registry	US-CDT (EKOS^TM^) + AC	489	High (5%); Int-high (95%)	24–48 h change in RV/LV ratio, other long-term	No
APEX-AV	2024	PCS	MT (AlphaVac^TM^) + AC	122	Int-high (100%)	48 h change in RV/LV ratio	Yes
PEERLESS	2024	RCT (head-to-head)	MT (FlowTriever®) + AC vs. any catheter-based + AC	550	Int-high (100%)	^f^Hierarchical WR composite assessed at 7 days or hospital discharge	No

Key studies include prospective cohorts supporting 510(k) authorization, industry-sponsored post-market prospective registries, and all randomized controlled trials (RCTs); other cohort studies (mainly retrospective) have also been completed but are not included here.

^a^References (chronological): ULTIMA (15); SEATTLE II (14); OPTALYSE-PE (not cited in manuscript; *JACC: Cardiovascular Interventions* 2018); FLARE (10); EXTRACT-PE (11); SUNSET sPE (not cited in manuscript; *JACC: Cardiovascular Interventions* 2021); RESCUE (12); CANARY (33); Kroupa (34); FLASH (45); STRIKE-PE (56); KNOCOUT-PE (44); APEX-AV (13); PEERLESS (not cited in manuscript; *Circulation* 2024).

^b^Year of publication of primary results.

^c^Total number of patients included in the study.

^d^Some percentages are estimates based on reported percentages of cardiac biomarkers.

^e^Criteria were RV/LV ratio reduction 25% by 48 h, systolic pulmonary artery pressure reduction by 30% or ≤35 by 24 h, and reduction of Qanadli score by 30% by 48 h.

^f^Composite of (i) all-cause death, (ii) intracranial haemorrhage, (iii) major bleed, (iv) clinical deterioration/bailout, and (v) ICU admission and length of stay.

AC, anticoagulation; CDT, catheter directed thrombolysis; US-CDT, ultrasound assisted catheter directed thrombolysis; ESC, European Society of Cardiology; int, intermediate; MT, mechanical thrombectomy; PCS, prospective cohort study; RCT, randomized control trial; RV/LV, right ventricle/left ventricle; trop, cardiac troponin; NT-proBNP, N-terminal prohormone of brain natriuretic peptide; WR, win ratio.

## Evidence and outstanding clinical questions for endovascular therapy in intermediate-risk PE

**Figure 2:**
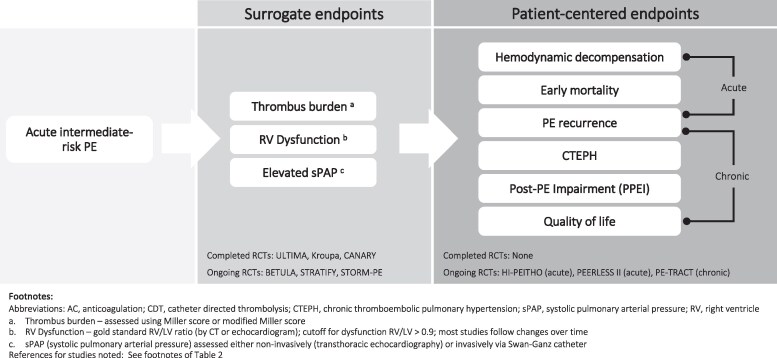
Schematic of clinical endpoints in completed and ongoing randomized controlled trials (anticoagulation-controlled) in intermediate-risk pulmonary embolism.

The body of key completed and ongoing studies of endovascular therapies in intermediate-risk PE (primarily intermediate-high risk) includes prospective cohorts and RCTs (*Table 1* and *Figure 1*). Two forms of RCTs exist (*Figure 1*): those comparing adjunctive endovascular modalities head-to-head (not discussed here), and those comparing adjunctive endovascular therapy with anticoagulation alone.^29^ The trials with anticoagulation control arms can be further stratified by use of either surrogate or patient-centred endpoints (*Figures 1* and *2*). Collectively, these studies have demonstrated that endovascular therapies improve short-term surrogate measures of effectiveness such as RV dysfunction.^4,29^ However, over a decade removed from the first device authorization in the USA, several key clinical questions remain unanswered.

### Outstanding clinical question 1: surrogate endpoint validity

Among haemodynamically stable patients with acute PE, RV dysfunction has been associated with short-term mortality.^30,31^ Measures of RV dysfunction—most commonly the RV/LV ratio—were adopted as surrogate markers of effectiveness in clinical research, beginning with studies of systemic fibrinolysis followed by endovascular devices. However, the epidemiologic relationship between RV dysfunction and short-term mortality does not guarantee that rapid improvements in RV dysfunction will translate to improvements in mortality. Indeed, this limitation is explicitly acknowledged in the 2019 AHA Scientific Statement on interventional therapies for acute PE.^4^

### Outstanding clinical question 2: surrogate benefit of endovascular therapy

It remains unclear whether the addition of endovascular therapy to anticoagulation improves RV dysfunction—in the short or long term—beyond the improvements seen with anticoagulation alone.^27,32^

To date, only three relatively small RCTs have addressed this question (*Figure 1*) with mixed results. In ULTIMA (*N* = 59), the addition of EKOS^TM^ improved the RV/LV ratio more than anticoagulation alone at 24 h, but by 90 days improvements were similar in both groups.^15^ In CANARY (*N* = 94), the addition of CDT to anticoagulation led to no difference in the primary outcome of RV/LV ratio > 0.9 at 3 months. However, this study was stopped prematurely during the COVID-19 pandemic, and the secondary outcome of 72 h improvement in RV/LV ratio favoured the CDT arm.^33^ In a pilot RCT by Kroupa (*N* = 23), the addition of CDT resulted in significant 48 h reduction in RV/LV ratio, but longer term results are unavailable.^34^

Three ongoing RCTs comparing various endovascular devices with anticoagulation only controls (BETULA [NCT03854266]; STRATIFY [NCT04088292]; STORM-PE [NCT05684796]) will also address this question (*Figure 2* and *Table 2*).

**Table 2:** Clinical endpoints in completed and ongoing RCTs (AC-controlled) of endovascular therapy in intermediate-risk PE

Study^a^	*N* ^b^	Intervention	Control	Efficacy or Effectiveness^c^	Safety^c^	Status^b^	Notes
*Complete*							
ULTIMA	59	US-CDT (EKOS^TM^) + AC	AC alone	Primary: 24 h change in RV/LV ratio	Secondary: death, major/minor bleeding, and recurrent VTE at 90 days	Published 2013	proBNP not reported; 304/363 failed screening (125 due to clot location)
Kroupa et al.	23	CDT (Cragg-McNamara) + AC	AC alone	Primary: 48 h RV function improvement (three parameters)	Secondary: intracranial or life-threatening bleeding at 72 h (BARC 3c or 5)	Published 2022	
CANARY	94	CDT (Cragg-McNamara) + AC	AC alone	Primary: 3 month RV/LV ratio > 0.9	Secondary: major bleeding at 72 h (BARC 3 or 5)	Published 2022	Terminated early (COVID-19)
*Ongoing*							
HI-PEITHO	544	US-CDT (EKOS^TM^) + AC	AC alone	Primary: 7 day composite: PE-mortality, PE-recurrence, and decompensation	Secondary: GUSTO major bleeding (7 day), ISTH major bleeding (7, 30, 180 day), serious adverse events, stroke	Start 8/2021 Completion 8/2026	Higher risk patients^d^; Decompensation includes NEWS score
PE-TRACT	500	Any (operator choice) + AC	AC alone	Primary: 3 month PVO_2_ by CPET; 1 year NYHA functional classification	Primary: 7 day ISTH major bleeding	Start July 2023 Completion January 2028	Funded by NHLBI (only government-funded trial); Operator selects device; Primary safety endpoint
PEERLESS II	1200	MT (FlowTriever®) + AC	AC alone	Primary: Composite win-ratio of deterioration/bailout (discharge or 30 days), all-cause readmission (30 days), and dyspnoea at 48 h	Secondary: Major bleeding (BARC 3b, 3c, 5a, or 5b) at 30 and 90 days	Start November /2023 Completion July 2026	Higher risk patients^e^; Complicated primary efficacy endpoint
BETULA	60	Low-dose CDT (Uni-Fuse) + AC	AC alone	Primary: 24 h change in RV/LV ratio	Secondary: Major and minor bleeding (not defined further)	Start March 2020 Completion March 2024	No updates past estimated completion date; Denmark
STRATIFY	210	US-CDT (EKOS^TM^) + AC or low-dose systemic thrombolysis + AC	AC alone	Primary: 48–96 h change in thrombus burden (Miller score)	Secondary: TIMI major and minor bleeding (by discharge)	Start June 2019 Completion October 2024	Three parallel arms; Denmark
STORM-PE	100	MT (Indigo®) + AC	AC alone	Primary: 48 h change in RV/LV ratio	Secondary: 7 day major adverse events	Start November 2023 Completion October 2026	

^a^References (completed studies): ULTIMA (15); CANARY (33); Kroupa (34); References (ongoing studies): BETULA (NCT03854266); STRATIFY (NCT04088292); STORM-PE (NCT05684796); HI-PEITHO (38); PEERLESS II (39); PE-TRACT (40).

^b^Estimated for ongoing trials from clinicaltrials.gov, extracted November 2024; completion refers to estimated date of study completion (last data collection for primary endpoints).

^c^Secondary efficacy endpoints and other safety endpoints are not included in this table but available online at clinicaltrials.gov.

^d^Elevated risk of deterioration defined by two of the following three criteria (i) ECG-documented tachycardia with heart rate ≥100 beats per minute, not due to hypovolemia, arrhythmia, or sepsis; (ii) SBP ≤ 110 mmHg for at least 15 min; (iii) respiratory rate > 20 × min^−1^ or oxygen saturation on pulse oximetry (SpO_2_) < 90% (or partial arterial oxygen pressure < 60 mmHg) at rest while breathing room air.

^e^At least one measure in two of three separate categories: (i) Haemodynamic: (a) SBP 90–100 mmHg, (b) Resting heart rate > 100 bpm; (ii) Biomarker: (a) Elevated cardiac troponin (troponin I or troponin T, conventional or high sensitivity), (b) Elevated BNP or NT-proBNP, (c) Elevated venous lactate ≥2 mmol/L; (iii) Respiratory: (a) O_2_ saturation < 90% on room air (b) Supplemental O_2_ requirement ≥ 4 L/min (c) Respiratory rate ≥ 20 breaths/min (d) mMRC score > 0.

AC, systemic anticoagulation; CPET, cardiopulmonary exercise testing; MT, mechanical thrombectomy; PVO_2_, peak oxygen consumption; NEWS, National Early Warning Score (established by Royal College of Physicians) is an aggregate scoring system based on physiological measurements—respiration rate, oxygen saturations, supplemental oxygen need, temperature, systolic blood pressure, heart rate, and level of consciousness; NHLBI, National Heart, Lung, and Blood Institute; US-CDT, ultrasound-assisted catheter-directed thrombolysis.

### Outstanding clinical question 3: patient-centred benefit of endovascular therapy

The most important question is whether endovascular therapy added to anticoagulation improves clinical outcomes for patients, including mortality, haemodynamic deterioration, symptom resolution, and sequalae that contribute to long-term morbidity such as chronic thromboembolic pulmonary hypertension (CTEPH), post-PE impairment (PPEI), or recurrence of disease (*Figure 2*).^4^ In intermediate-high risk PE, short-term mortality ranges from 1–3% in clinical trials^26,27^ to 3–15% in observational studies,^4,20^ CTEPH develops in 2–3% of patients, and post-PE impairment (PPEI) in 10–30%.^35–37^

Although no RCT has studied such patient-centred outcomes, three large ongoing RCTs are doing so (*Table 2*). The HI-PEITHO and PEERLESS II trials, funded by Boston Scientific and Inari Medical, respectively, are currently enrolling intermediate-high risk patients who are also classified at elevated risk of decompensation—leveraging criteria derived post-hoc from PEITHO^28^—to endovascular therapy plus anticoagulation or anticoagulation alone. Both trials use short-term composite primary efficacy endpoints. The HI-PEITHO study will assess 7 day PE-related mortality, PE recurrence, and haemodynamic collapse,^38^ whereas the primary endpoint in PEERLESS II is a hierarchical composite win ratio of 30 day all-cause mortality, clinical deterioration, readmission, need for bailout therapy, and 48 h dyspnoea severity.^39^ Both of these studies will also examine other secondary outcomes important to patients, such as hospital and intensive care unit (ICU) length of stay. Finally, the publicly funded PE-TRACT study is randomizing intermediate-risk patients with symptomatic proximal PE and RV/LV > 1 to any endovascular intervention (operator choice) in addition to anticoagulation or anticoagulation therapy alone, with long-term functional primary endpoints^40^ of peak oxygen consumption at 3 months and New York Heart Association functional classification at 1 year. Results of all three trials are expected before the end of the decade (*Table 2*).

### Outstanding clinical question 4: safety of endovascular therapy

Safety data are paramount in comparing adjunct procedural management with medical management alone, as procedures introduce additional risk. Some endovascular therapies, like EKOS^TM^ US-CDT and traditional CDT, aim to reduce the bleeding risk of systemic thrombolysis by locally delivering smaller doses. Mechanical thrombectomy systems (e.g. FlowTriever®, Indigo®, and AlphaVac^TM^) avoid thrombolytics but introduce larger-bore catheters and various aspirating mechanisms that carry their own risks, including bleeding and rare, but severe structural complications.^41,41–43^

The three relatively small RCTs of endovascular therapy were not powered to assess safety, and most prospective studies have reported low (1–3%) major adverse event rates,^10–15,33,34,44,45^ comparable to major bleeding rates with anticoagulation alone in the control arm of PEITHO and a subsequent multicentre European prospective study of anticoagulation alone (PEITHO-2).^26,27^ In pooled analyses including larger observational studies that reflect real-world use, major bleeding with CDT has been found both comparable to^46^ and higher than^47^ with AC alone, but lower than with systemic thrombolysis.^47^ Additionally, a recent real-world claims-based analysis found much higher rates of major bleeding with endovascular therapy than previously described (10–15%).^41^ These discrepancies underscore the importance of adequately powered randomized studies and robust post-approval comparative safety data analyses to elucidate the true safety profile of endovascular therapies. The PE-TRACT study includes a primary endpoint of major bleeding at 7 days, and HI-PEITHO and PEERLESS II will report bleeding and other complications as secondary outcomes (*Table 2*).^38–40^

## Drivers of endovascular device utilization in intermediate-risk PE

The paucity of comparative evidence is reflected in major American and European scientific statements and guidelines, where there are currently no Class I recommendations for endovascular therapy.^3,4,18^ The ESC guidelines, for example, recommend systemic anticoagulation for all intermediate-risk PE (Class I, Level A), reserving reperfusion for patients who deteriorate. In such cases, catheter-based interventions are recommended (Class IIa, Level C) only as an alternative to rescue thrombolysis (Class I, Level B).^3^

Nevertheless, endovascular therapy utilization in intermediate-risk PE has risen dramatically in the past decade.^6,7,9,48^ Between 2015 and 2022, 26.4% of intermediate-risk patients who triggered consults at Pulmonary Embolism Response Team (PERT) centres—where device use rates are generally higher—received endovascular therapy.^5^ Likewise, within the limitations of classifying severity (‘intermediate-risk’) using electronic medical record codes, a large retrospective analysis of the national readmissions database demonstrated that between 2017 and 2020 about 20% of intermediate-risk patients were managed with endovascular modalities.^8^

In the absence of new high-quality clinical evidence or changes in professional society recommendations, plausible drivers of utilization include clinical uncertainty, the emergence and effect of PERTs, limitations in regulatory and reimbursement mechanisms, the marketing of observational research, and financial incentives for various stakeholders.

### Clinical uncertainty

The top priority for physicians managing intermediate-risk PE is to prevent haemodynamic deterioration and death (*Figure 2*). Though uncommon in intermediate-risk PE, these outcomes still do occur. With increasing availability of endovascular tools, the tendency to offer up-front adjunct endovascular therapy reflects, in part, the limitations of contemporary risk stratification tools in predicting those who will go on to deteriorate. Current uncertainty is exemplified by inclusion criteria of ongoing RCTs—some of which were derived post-hoc from PEITHO—designed to enroll intermediate-high risk patients who are also at an elevated risk of deteriorating (*Table 2*).^28,38,39^ The evidence from ongoing randomized trials will be pivotal in optimizing patient selection for endovascular therapy in intermediate-risk PE.

### The advent and effect of pulmonary embolism response teams (PERTs)

Pulmonary embolism response teams emerged in the 2000s at specialized centres in response to the need for a timely, multidisciplinary approach to managing patients with life-threatening PE. Since their conception, PERTs have gained significant popularity. The institutional presence of a PERT has been associated with improvements in multiple parameters including proper risk stratification and triage (i.e. use of echocardiography and biomarkers of cardiac dysfunction), time to diagnosis and treatment, and short- and long-term multidisciplinary care for this complex condition.^49^ The ESC guidelines recommend consideration of PERT use in high-risk and select intermediate-risk cases (Class IIa, Level C),^3^ as these patients often require prompt clinical decisions and mobilization of resources. However, PERT programs have also been associated with sudden and sustained increases in utilization of endovascular therapies,^49,50^ particularly among intermediate-risk patients,^51^ and there is a paucity of data examining the differential impact of PERT implementation on clinical outcomes in high-risk vs. intermediate-risk PE.

### Regulatory and reimbursement mechanisms

Each of the five commercially available endovascular devices authorized for PE treatment came to the US market through the FDA’s pre-market notification 510(k) clearance process, which allows device authorization based on ‘substantial equivalence’ to an existing (predicate) device.^52,53^ Though the 510(k) pathway promotes innovation by reducing the time and cost of commercialization compared to the more rigorous premarket approval pathway, the lower levels of required clinical evidence permit manufacturers to focus on marketable innovations that can yield premium prices and reimbursement before said innovations have demonstrated improvements in hard outcomes.^52^ Indeed, in 2011, after reviewing the 510(k) clearance process at the request of the FDA, the Institute of Medicine determined that, with some exceptions, it ‘is not intended to assess safety and effectiveness of devices’.^53,54^

In the USA, following FDA 510(k) marketing clearance, insurers independently determine if they will cover the device or procedure in which the device is used. The Centers for Medicare and Medicaid Services (CMS) is the US’ largest insurer and its decisions often influence those of other public and private payers. In 2021, CMS controversially^55^ ended its national non-coverage policy for pulmonary embolectomy devices, allowing coverage determinations to be made locally by Medicare administrative contractors (MACs),^55^ who are mostly private insurers with heterogeneous processes. This decision has likely accelerated coverage, and in turn, accelerated adoption of endovascular therapies. Furthermore, because CMS made this decision instead of requiring ongoing evidence generation through its coverage with evidence development (CED) mechanism, the timing of high quality evidence was also affected.^55^

### Marketing of observational research

After crossing the regulatory hurdle, device manufacturers turn more attention toward marketing and sales. In the absence of high-quality clinical evidence, devices are promoted based on technological features and observational research. The most common examples are industry-sponsored post-market registries, such as the FLASH (FlowTriever®, Inari), KNOCOUT-PE (EKOS^TM^, Boston Scientific), and STRIKE-PE (Indigo®, Penumbra) (*Figure 1*).^44,45,56^ The limitations of these studies are acknowledged by investigators,^44^ some of which include selection bias and the omission of a parallel arm of patients treated with anticoagulation—precluding comparative effectiveness or safety analyses. Still, their results are frequently referenced in marketing material to promote both effectiveness and safety^57,58^ alongside unique device features such as bench-top associations between ultrasound power and clot weight reduction (Boston Scientific),^59^ self-expanding nitinol disks (Inari),^60^ or a computer feedback interface (Penumbra).^61^

While observational studies can be helpful in a variety of ways, including establishing absolute safety profiles, describing technical success, and describing real-world practice patterns, RCTs remain the gold standard for evaluating the comparative efficacy and safety of adjunct interventions.

### Financial incentives

Physicians may receive research payments, but also general payments from manufacturers, which include food and beverage, lodging, and consulting fees. On aggregate, medical device companies allocate a significant fraction of revenue (1.7%) to physician payments, and cardiologists receive more payments than most other specialties.^62^ Although no study has specifically examined industry payments to physicians and PE device use, payments—of any magnitude—are associated with use of therapies being marketed.^62,63^ Until its recent acquisition by Stryker, Inari Medical was a relatively young company that derived two-thirds of its revenue from FlowTriever® sales.^64^ Since its initial public offering in 2020, the company had rapidly increased its volume and magnitude of general physician payments. In 2020, it made 3500 general payments to physicians amounting to $913 700.^65^ Over the next three calendar years (2021, 2022, and 2023), these figures increased to 10,281, 19,910, and 38 027 general payments for $2.49 M, $4.86 M, and $6.74 M,^65^ respectively, representing 0.9%, 1.2%, and 1.4% of top-line revenues.^66^

Hospitals in the USA are reimbursed prospectively based on medical severity diagnosis-related groups (MS-DRGs). Medicare recalibrates DRG reimbursement rates annually; when new devices or procedures are adopted, prices (set by industry) raise average costs and reimbursement rises in parallel. Between 2021 and 2022, for example, the reimbursement for endovascular therapy for PE with major comorbidity or complications (MS-DRG 166) nearly tripled from $9040 to $24 550.^67^ Accordingly, an interventional PE service line can generate high revenues for a hospital system. Profits are challenging to estimate because financial figures such as device purchasing agreements and hospital costs are not publicly available. Without disclosing the price of its device, in its investor report, Inari Medical graphically promotes the profit margin hospitals received in 2023 using DRG-based reimbursement rates ($13 219 to $33 228).^64^

## Scenarios and considerations on the horizon in intermediate-risk PE

Fortunately, many outstanding clinical questions will be answered by the end of the decade by the three ongoing RCTs (PE-TRACT, HI-PEITHO, and PEERLESS II) which incorporate proper controls and patient-centred measures of efficacy.^38–40^ Depending on what these trials find, many possible scenarios occupy the horizon, a few of which are explored here.

### Scenario A: benefit with acceptable risk

The first scenario is that endovascular therapy is found to provide significant short- and/or long-term benefit, without significant safety risks compared to anticoagulation alone. Should this occur, endovascular therapies may gain strong recommendations for use in appropriate subsets of intermediate-risk patients. For example, if both trials examining short-term efficacy (HI-PEITHO and PEERLESS II) demonstrate a favourable risk–benefit profile for endovascular therapy, current risk stratification paradigms could evolve to reflect the novel inclusion criteria used in both of these studies (*Table 2*) designed to select intermediate-risk patients at elevated risk of deterioration (e.g. tachycardia, tachypnoea, hypoxia, low-normal systolic blood pressure). Subsequent studies would focus on cost-effectiveness as well as real-world safety free of the exclusion criteria in clinical trials.

### Scenario B: benefit with unacceptable risk

One or more of these trials may demonstrate benefits that are outweighed by harms including major bleeding (as with PEITHO) or other procedural risks associated with cardiac catheterization. Depending on relative effect sizes, endovascular therapies may be recommended, particularly for patients who clinically deteriorate on anticoagulation alone, as seen with systemic thrombolysis in intermediate-risk PE following the results of PEITHO.^3^ Recommendations may narrow to specific patient populations, and subgroup analyses from these large and well-conducted RCTs could facilitate the development and validation of clinical tools to help with early identification of patients with favourable risk–benefit profiles.

### Scenario C: no benefit

Finally, these RCTs could fail to demonstrate any short-term (HI-PEITHO, PEERLESS II) or long-term (PE-TRACT) benefits of endovascular therapy in intermediate-risk PE. This scenario will put evidence-based medicine at odds with market forces that have resulted in contemporary levels of utilization. Given the clear superiority of these trials relative to predecessors by virtue of size, controls, and endpoints, this scenario would lend toward a potential medical reversal, or de-adoption of endovascular therapy in intermediate-risk PE.^17^

### Other considerations

As the evidence for endovascular therapy in intermediate-risk PE matures, other dimensions of PE care—including therapeutics, diagnostics, and our understanding of the disease and its sequalae—will evolve in parallel.

Within the therapeutic realm, the PEITHO-3 trial is randomizing 650 intermediate-high risk patients satisfying additional criteria for ‘elevated risk’—resemblant of criteria used in both HI-PEITHO and PEERLESS II—to receive systemic, dose-reduced alteplase (a synthetic thrombolytic medication) in addition to standard anticoagulation.^68^ The primary endpoint is a hard composite of all-cause mortality, deterioration, and PE recurrence by 30 days. A favourable risk–benefit profile for dose-reduced alteplase may lead to recommendations for its use in intermediate-high risk PE—providing treatment alternatives for patients in resource-limited settings, or those who simply prefer medical management. Depending on the results of anticoagulation-controlled endovascular RCTs happening in parallel, this may also set the stage for comparative cost-effectiveness analyses or even a head-to-head trial of endovascular therapy against dose-reduced alteplase in intermediate-high risk PE.

On the diagnostic front, several artificial intelligence (AI) solutions have already received FDA 510(k) clearance for PE diagnosis and RV/LV analysis for faster diagnosis and risk stratification. Exciting long-term capabilities of AI include the potential to identify prognostically important radiographic patterns too complex for human recognition, as demonstrated with chest radiographs and cardiovascular risk.^69^

Finally, though only a small fraction of intermediate-risk patients go on to develop CTEPH, a much larger percentage report post-PE impairment (PPEI)—characterized by persistent dyspnoea and functional impairment—which remains poorly understood but will gain clarity with high-quality functional and outcome data from ongoing RCTs.

## Conclusion

The contemporary management of intermediate-risk PE presents a complex interplay between a dynamic disease, timing of clinical evidence, regulatory and reimbursement mechanisms, marketing, and financial incentives across the health care value chain. Contrary to what procedural momentum would suggest, the evidence-based medicine community is at a crossroads. Cautious optimism is warranted as we patiently await robust RCT evidence, and continued enrollment in these RCTs for appropriate patients is critical to finally answer the myriad outstanding questions and optimize outcomes for the large and heterogenous group of patients with intermediate-risk PE.

## Data Availability

The financial data underlying this article will be shared on reasonable request to the corresponding author.

## Lead author biography

At the time of this publication, Dr. Shahriar was a resident in internal medicine at the University of Chicago. He plans to pursue fellowship training in cardiology and critical care.
